# The Hospital Officer's Bookshelf

**Published:** 1912-10-05

**Authors:** 


### THE HOSPITAL OFFICER'S BOOKSHELF.

A Handbook for Nurses. By J. K. Watson, M.D. Fifth Edition, completely revised. (London : The Scientific Press. Pp. 518. Price 5s. net.)

This well-known manual pursues an even tenor of new editions, undisturbed by the steadily increasing competition which it has to face. In this fifth edition the work of revision has been unusually complete, and, in fact, a good part of the book has been rewritten altogether. Dr. Watson's handbook does not set out to teach nurses and probationers the elements of nursing itself; but rather to supply them with that general outline of medical and surgical science which it is advisable and necessary that they should comprehend. Incidentally, of course, many details and principles of nursing are touched upon and illustrated; for nursing and medicine are two interdependent sciences which have many points of contact.

In constructing a text-book of this aim and scope, the great difficulty is always to know what to leave out, and to express the root of the matter in hand without undue technicality and bewildering detail. This difficulty Dr. Watson very successfully overcomes, and probably this is one of the reasons for the great popularity of his book among nurses. He is certain at no distant date to be required to edit yet another edition; when that time comes there are a few small points which would be worth attending to. The sections on anæsthetics should be revised by an anæsthetist. This applies to the remarks on local anæsthetics as well as to the section on general anæsthesia. The management of opium poisoning needs revision. Schäfer's method of artificial respiration should be mentioned.

The Art of Dispensing. By Peter MacEwan, F.C.S. Ninth Edition. (London : Offices of *The Chemist and Druggist.* 1912. Price 6s.)

Dispensing as an art is, we fear, not in that flourishing state that it was thirty years ago. Many causes are responsible for this, such as the enormous increase in the amount of dispensing done by the doctors themselves and the growth of "tabloid" medication. Nevertheless, the science and art of compounding drugs loom very large in the curriculum of the budding pharmacist, and will no doubt continue to occupy a big slice of his hours of study. Anyone familiar with the dispensing methods of the average practitioner must be filled with awe at the superior methods of the first-class pharmaceutical chemist, though whether the patient derives any greater benefit from the medicine dispensed with meticulous accuracy than from that put together by the doctor is open to doubt. Perhaps in the future there will be seen still further advances towards that prophetic utterance about "throwing physic to the dogs." But, at any rate, that time is not yet, and the pharmaceutical student must go through a somewhat arduous but not uninteresting course of pill-making and prescription-compounding, including the solution of caligraphic puzzles and impossible chemical combinations. The ignorance and carelessness of many prescribers provide food for much thought on the part of the unfortunate pharmacist, and afterwards he revenges himself on the coming generation by setting these undecipherable incompatibilities to examination candidates. What happened to the patients does not, unfortunately, transpire, probably because the doctor "lost sight of them." In this volume, published by our contemporary *The Chemist and Druggist,* we have very clearly set out the methods and processes involved in compounding medical prescriptions. The text is nothing if not practical, and for elucidating difficult points we can imagine no more satisfactory guide. No inconsiderable portion of the book is occupied by notes on new and unofficial remedies, appendices of foreign terms, test-prescriptions, and other useful memoranda. The present ninth edition has been considerably amended, and its usefulness thereby increased, notably by the inclusion of a section on the dispensing and filling of ampoules.

The Nurses' Complete Medical Dictionary. By M. Theresa Bryan. (London : Baillière, Tindall and Cox. 1912. Pp. 196. Price 2s. net.)

This pocket dictionary is very similar in size, style, and scope to the well-known "Nurses' Pronouncing Dictionary," by Miss Honnor Morten, which has run through seven editions and has long been a favourite with nurses. So obvious, indeed, is the challenge that comparisons, odious or otherwise, are bound to be suggested to the minds of those who teach nurses and advise them what books to buy. This newcomer in the field certainly has advantages in the matter of type; but these are counterbalanced by the handier size (for a pocket volume) and lighter weight of the established favourite. As regards the definitions, there is not much to choose between the two; both are good, and the distinct family resemblance between them is no proof that the new one has been plagiarised from the old. In the selection of the words given we award pre-eminence to the "Nurses' Pronouncing Dictionary," the last two editions of which have been purged of the numerous obsolete words which disfigured the earlier editions and which duly appear (with some even more obsolete and grotesque still) in Miss Bryan's volume. In a word, the latter is attempting to fill a place already adequately occupied. What measure of success she may attain in ousting Miss Morten remains to be seen; but the competition can only be regarded as eminently satisfactory from the point of view of the nursing profession, since it is bound to result in increased efficiency in a field where hitherto they have had no choice.

Manual for Women's Voluntary Aid Detachments. By Lieut.-Col. P. C. Gabbett, M.R.C.S. (Bristol : John Wright and Sons. 1s. net.)

This little manual is one which no woman who has joined a "V.A.D." should omit to possess and study. It is sensible and sane and practical from cover to cover, full of just that which it most concerns the V.A.D.s to comprehend. The two points which strike one most forcibly on perusing it are these : that Colonel Gabbett evidently anticipates at no distant date the invasion of this country, and is under no delusion as to the horrible scenes that such a conflict will entail; and that if the Medical Service of the Territorial Force is not to remain a mockery, the War Office must provide the V.A.D.s with proper equipment instead of leaving it to the private effort and subscriptions of the members themselves. Besides those to whom the booklet is specifically addressed, there are two classes who might profit by reading this book : one is the boastful jingo, ready to provoke war on any pretext; the other is the no-Navy peace-at-any-price person who has been so prominent of late years in England.

